# Obituary: remembering professor Arkadiusz Kozubek (1947–2016)

**DOI:** 10.1186/s11658-016-0010-4

**Published:** 2016-09-08

**Authors:** Aleksander F. Sikorski, Jerzy Gubernator

**Affiliations:** 1 Departments of Cytobiochemistry and Lipids and Liposomes, University of Wrocław, F. Joliot-Curie 14a, 50-383 Wrocław, Poland; 2 Lipids and Liposomes, Faculty of Biotechnology, University of Wrocław, F. Joliot-Curie 14a, 50-383 Wrocław, Poland

Professor Arkadiusz Kozubek, a talented, creative biochemist and membrane biologist working on plant phenolic lipids (mostly alkyl resorcinols and liposomes), best known for his many contributions to developing methods in phenolic lipids analysis and their effect on animal organisms, sadly passed away on 20 May, 2016, at the age of 69. His loss will be deeply felt by the research community that benefitted profoundly from his work over the past four decades, and from their rewarding personal interactions with him over his career. Professor Kozubek was among three of the founding editors of Cellular and Molecular Biology Letters, who established the journal in 1996. Apart from working on the daily editorial issues, he was also responsible mainly for the graphics of the first generation of the journal (1996–2005) and for the contacts databases. Arkadiusz Kozubek finished his Master’s education and his Ph.D. in biochemistry from the University of Wrocław in 1975 and he worked here for most of his life. In the late seventies and early eighties he made several post-doctoral stays at the University of Utrecht where he worked with Professors Ben de Krujif, H. de Gier and R. Demel. In 1985 he spent a year in Delaware working with Prof. M. K. Jain. In the early nineties he co-worked with Prof. J. Tyman in London. Almost all his professional life was connected with work on phenolic lipids. This, we believe, is the field he really loved. He published more than 100 papers throughout his career and he participated in several conferences in which he was invited to lecture. His other field of interest was the use of liposomes as drug carriers. He and his younger colleagues patented several formulations and methods in this area. He organized several conferences on liposomal drug carriers which were all very successful.

Arek was a great teacher. Many students performed their Master theses under his expert care and guidance, and 15 successfully finished their Ph.D. thesis under his supervision. Three senior scientists, who were his former Ph.D. graduates, are now appointed at the University of Wrocław, one being his scientific successor and head of his former Department. Many of his former students and collaborators now work in industry and academic research laboratories across Europe and the USA.

Arek was for several years a deputy director of the Institute of Biochemistry and was the director of this Institute for a period of six years. He was an extremely efficient and well-organized administrator, having still time for science and being keen to learn about the latest developments in science. Apart from that he was manually gifted, able to fix all the equipment in the lab, ranging from computers to the simpler equipment such as vacuum pumps to the more complex equipment such as the Nima balance, that were so important to his research. Most of the small and close scientific community that he worked with will remember him for this personal, knowledgeable and hands-on approach to everything that he did.

